# Parasite-induced aggression and impaired contest ability in a fish host

**DOI:** 10.1186/1756-3305-3-17

**Published:** 2010-03-15

**Authors:** V N Mikheev, A F Pasternak, J Taskinen, E T Valtonen

**Affiliations:** 1 Laboratory of Behaviour of Lower Vertebrates, Institute of Ecology and Evolution, Russian Academy of Sciences, 33 Leninskii pr, 119071 Moscow, Russia; 2 Laboratory of Plankton Ecology, Institute of Oceanology Russian Academy of Sciences, 36 Nakhimovskii pr, 117997 Moscow, Russia; 3 Department of Biological and Environmental Science, University of Jyväskylä, PL 35, 40351 Jyväskylä, Finland

## Abstract

**Background:**

Success of trophically transmitted parasites depends to a great extent on their ability to manipulate their intermediate hosts in a way that makes them easier prey for target hosts. Parasite-induced behavioural changes are the most spectacular and diverse examples of manipulation. Most of the studies have been focused on individual behaviour of hosts including fish. We suggest that agonistic interactions and territoriality in fish hosts may affect their vulnerability to predators and thus the transmission efficiency of trophically transmitted parasites. The parasite *Diplostomum spathaceum *(Trematoda) and juvenile rainbow trout, *Oncorhynchus mykiss*, were used to study whether infection can alter aggression rates and territorial behaviour of intermediate fish hosts.

**Results:**

The changes in behaviour of rainbow trout, *Oncorhynchus mykiss*, infected with an eye fluke *Diplostomum spathaceum *(Trematoda), was monitored over the course of an experimental infection for 1.5 months. At the beginning of their development, not yet infective *D. spathaceum *metacercariae decreased the aggressiveness of rainbow trout. By the time that metacercariae were fully infective to their definitive hosts, the aggressiveness increased and exceeded that of control fish. Despite the increased aggressiveness, the experimentally infected fish lost contests for a territory (dark parts of the bottom) against the control fish.

**Conclusions:**

The results obtained indicate that the parasitized fish pay the cost of aggressiveness without the benefit of acquiring a territory that would provide them with better protection against predators. This behaviour should increase transmission of the parasite as expected by the parasite manipulation hypothesis.

## Background

Success of trophically transmitted parasites depends to a great extent on their ability to manipulate their intermediate hosts in a way that makes them easier prey for target hosts [1,2]. A large range of host phenotypic traits can be altered by parasites, including morphology, physiology and behaviour. Behavioural changes are the most spectacular and diverse examples of manipulation [3,2,4]. Aggression and territoriality are important in foraging, mating, defense against predators and other vital activities [5,6]. More aggressive individuals could establish individual territories faster, where they are more protected from predators [7-9]. On the other hand, aggressive individuals could be less vigilant, more conspicuous and vulnerable for predators during contests [10,11]. These behaviours could strongly influence the probability of parasite transmission to the next host. However, no effect of the trematode *Telogaster opistorhis *on the competitive ability and aggression in male upland bullies *Gobiomorphus breviceps *(Pisces: Eleotridae) was found [12]. In a previous study [13] upland bullies infected with the same parasite demonstrated a reduced anti-predator response.

We used the parasite *Diplostomum spathaceum *(Trematoda) and juvenile rainbow trout, *Oncorhynchus mykiss*, as a model system to study whether infection can alter aggression and territorial behaviour of intermediate fish hosts. Juvenile *O. mykiss*, like many other salmonids, are often territorial and aggressive while living in streams and lakes [14-17]. *D. spathaceum *has a three-host life cycle [18]. It matures in the intestine of piscivorous birds (definitive hosts) following ingestion of an infected fish. The parasite metacercaria develop in fish eye lenses impairing host vision [19-21] and crypsis [22], and causing surface-seeking behaviour [23], reduced escape responses [24], and altered shoaling behaviour [25]. All these make infected hosts more vulnerable to predators and facilitate parasite transmission.

Individual territories of juvenile salmonids are contestable, because they contain not only profitable foraging sites [26], but also refuges against both aquatic and aerial predators [17,8]. Initial stages of a territory acquisition are most dramatic and costly and are accompanied by increased aggression [27,28]. Aggression, together with fish size and prior residency, is one of the major determinants for establishing an individual territory [29,15,30].

Both social interactions and territory-holding in animal hosts may affect host vulnerability to predators and thus transmission efficiency of trophically transmitted parasites. Infection could make fish less aggressive, which would prevent them from acquiring a territory. Or infection could make fish more aggressive which would allow them to establish a territory and benefit from a lower predation risk [15,6] and thus the decreased probability of transmission. However, aggressive behaviour during contests may make fish less vigilant and more conspicuous to visual predators [10,28]. In addition, before development of a parasite to an infective stage, exposure of the host to any predation would be disadvantageous. After achieving infectivity, host behaviours that increase predation risk (by the required next host) should benefit the parasite [31,2]. Thus, an ideal manipulator would increase aggressiveness of the host and decrease its success in fighting but not until the complete development of the parasite.

We tested the hypotheses on parasite adaptive manipulation related to host aggressiveness and territoriality by following behaviour of rainbow trout infected with *D. spathaceum *during the course of an experimental infection up to 48 days, as *D. spathaceum *become infective to the definitive hosts after 1-2 months development in fish [32]. We induced competition for a territory (dark substrate patch; [33,34]), recorded aggressive behaviours, substrate choice, and followed outcome of dyadic contests. Our prediction was that if *D. spathaceum *manipulates fish behaviour, we should find (1) reduced aggressiveness due to the parasite in early stages of infection, (2) increased aggressiveness when the parasite has reached infectivity to definitive hosts, (3) reduced ability to win a contest when fish are parasitized by infective metacercariae.

## Methods

### Fish, Parasites and Infection Procedure

Experimental work was carried out at Konnevesi research station, University of Jyväskylä, in July-September 2005. Juvenile rainbow trout, *Oncorhynchus mykiss *(mean length ± SE: 89.1 ± 1.6 mm), were used as fish hosts, because rainbow trout is highly susceptible to infection by trematode cercariae [35], easily cultured and maintained under laboratory conditions. Fish were obtained from a commercial fish farm. They contained a low number (mean ± SE: 5.72 ± 0.59 parasites fish^-1^) of naturally acquired *Diplostomum spathaceum*. Naturally infected (control) fish were compared with experimentally infected fish (mean number of parasites per fish ± SE: 87.9 ± 5.8 ind fish^-1^). Small numbers of *D. spathaceum *cercariae were acquired by both control and experimental fish in the course of experiment from the flow-through system. We refer to experimentally infected fish as "infected" and naturally infected as "control".

We infected randomly chosen fish by exposing them to *D. spathaceum *cercariae under laboratory conditions at 17°C in four 150 l plastic tanks. Trematode cercariae were obtained from 8 naturally infected *Lymnaea peregra *snails. The snails were allowed to produce cercariae for four hours. We pooled all cercariae into one suspension and counted the density of parasites in 10 1-ml samples. Infection dose was 200 cercariae per fish. Groups of 70 fish were exposed to parasites for 30 min in each of the four 150 l tanks used, and then fish were transferred to a 1000-l flow-through dark green holding tank. Three hundred control fish in the other four 150 l tanks were sham exposed with water and were separately kept in another 1000-l flow-through dark green tank. Fish were maintained under similar conditions (mimicked natural photoperiod, feeding twice a day with commercial food pellets of appropriate size) during the 48 days of experiments.

### Substrate Choice and Aggressive Behaviour

Four flow-through dark brown 180 l plastic tanks of 170 × 30 × 40 cm size each were used to observe social interactions and distribution of infected and control fish. Each tank was divided with partitions into three compartments. The two end compartments (70 × 30 × 40 cm) were separated from the central one (30 × 30 × 40 cm) by partitions with rectangular holes of 5 × 3 cm near the bottom and vertically sliding doors to control the passage from one compartment to another. The central compartment was used as a start chamber. The bottom of one of the randomly chosen end compartment and central compartment was covered with white plastic, while another end compartment was left dark brown. Young rainbow trout prefer dark substrates over which fish are more cryptic [22], and they demonstrate aggression while competing for such places [33,34].

Groups of 5 fish, either experimentally infected, or control, were placed into the central compartment and left for 15 minutes acclimation. Water temperature was kept at 16-17°C, illumination 150 lux. After acclimation, water flow was turned off and the sliding doors were gently opened. Fish were allowed to move freely between compartments for 3 hours. Number of fish in each compartment was recorded 5 times with 5-min intervals in three recording sessions: immediately, then 1 and 3 hours after release. This was done by two observers hidden behind screens with slots for observation. During a recording session, each observer monitored his/her own tank. Immediately after release fish froze immobile close to the bottom, then moved actively throughout the tanks. The aggressive behaviours observed included charge, chase, nip, and lateral display [14]. We did not count them separately but use the total number of acts of aggression as a response variable in the statistical analyses. Acts of aggression were counted not for an individual fish, but for the whole group of 5 fish. Each observer recorded fish distribution and aggression in two tanks (one after another) in which fish were released with a 15-min time lag. Aggression rate was assessed as a total number of acts of aggression per 30 min (15 min count after the end of the 2^nd ^plus 15 min count after the end of the 3^rd ^recording sessions). Aggression interactions occurred almost exclusively in the dark-bottom compartment.

Twelve trials, 6 with randomly chosen infected and 6 with control fish were done per day. They were repeated the following day, so the total number of replicates per a certain post-infection date was 12 for infected and 12 for control fish. We assessed aggression and substrate preference 4 times: on the day of infection, 7, 30 and 48 days post-infection (PI). At the end of each test fish were transferred to separate holding tanks and were not used in the following tests. After each test the fork length of anaesthetized infected and control fish was measured. On days 7 and 48 PI the anaesthetized and killed fish were inspected for the number of parasites in the eye lenses (Table 1), and on day 48 PI also weighed.

**Table 1:** Fish size and intensity of *Diplostomum spathaceum *infection in the experiment on aggression and substrate choice

Parameters	Before infection	Days post-infection					
		
		7		30		48	
		
		Control	Experiment	Control	Experiment	Control	Experiment
Fork length, mm	89.1 ± 1.8 (30)	94.2 ± 1.4 (20)	95.6 ± 1.3 (20)	114.3 ± 2.1 (20)	113.4 ± 2.5 (20)	122.1 ± 2.0 (30)	121.5 ± 2.2 (30)

Number of metacercariae	5.7 ± 0.6 (30)	6.8 ± 0.7 (20)	82.5 ± 6.6 (20)	not checked	not checked	8.2 ± 0.4 (30)	87.9 ± 5.8* (30)

Means ± SE are given, n - in brackets. * - intensity of experimental infection could be overestimated, because experimentally acquired parasites became similar in size to those acquired before experiment.

### Dyadic Contests

We tested the ability of fish to establish an individual territory over a dark substrate in dyadic contests with one infected and one control fish. Since *D. spathaceum *metacercariae become infective for the final host after 1-2 months development in fish [32], these contests were conducted 40 days post-infection, at the beginning of intensive cataract formation [21]. Two randomly chosen fish from infected and control groups were simultaneously released into the central compartment of the testing tanks. After 15 min acclimation the sliding doors were gently opened and fish allowed to move freely between compartments. After a period of intensive swimming (10-60 min), when fish often visited all the three compartments, preference for the dark compartment gradually increased. This was accompanied by aggressive interactions, which occurred almost exclusively over the dark substrate. Most of the interactions were asymmetric and one of the fish eventually became a single holder of the dark-bottomed compartment, not allowing another fish to enter the compartment for more than 10-20 s. When this lasted for a period over 15 min, we considered the holder of the dark compartment a winner of the contest. If the winner was not determined within 3 hours, the contest was terminated and the result was rated as a "draw". Sixteen dyadic contests were carried out within 2 days. The fish were afterwards anaesthetized using 0.01% of MS 222, killed, measured, weighed and inspected for the number of parasites in the eye lenses.

To check whether the outcome of dyadic contests is influenced by the number of metacercariae in eye lenses of the control fish, they were tested in randomly chosen pairs in the same way as described above. Twelve dyadic contests were carried out within 2 days. The fish were afterwards anaesthetized using 0.01% of MS 222, killed, measured, weighed and inspected for the number of parasites in the eye lenses.

### Data Analysis

Data on aggression rates and substrate choice, i.e. distribution of infected and control fish between the compartments were log (x+1) transformed to meet the assumptions of parametric tests. Two-tailed tests were used. The effect of infection status (fixed effect; experimentally infected vs. control) and time (fixed effect, 1, 7, 30 and 48 days) on aggression rate (sum of different aggressive acts), and on substrate choice (number of fish in each compartment after 1 and 3 hours) were studied using 2-way ANOVA with Fisher LSD post-hoc tests. The results of dyadic contests between infected and control fish were analyzed with Chi square test. Differences in fish size distributions between groups were analyzed with Mann-Whitney U test.

### Ethical Note

To study the effects of metacercariae of the same age on the behaviour of the host, we used experimentally infected fish. The level of experimental infection (mean number of parasites per fish ± SE: 87.9 ± 5.8 ind fish^-1^) was within the limits of naturally occurring parasite load (up to 200-500 ind fish^-1^, [36,37]). Level of mortality of infected fish in our experiments was low (less than 1% per month) and did not exceed that of control fish. By the end of experiment the size and weight of control and infected fish did not differ. This shows that feeding rates of experimentally parasitized fish was not reduced. No visible damages to fish were observed in the contests. We minimized the required number of animals that were killed and dissected. All fish that were used in distribution and aggression level trials and not killed for infection assessment were afterwards transferred to separate holding tanks for use in other experiments not related to the topic of this paper. Fish size distributions and infection levels were checked at the beginning and at the end of experiments. These fish and those from the dyadic contests were killed at the end of the tests with 0.01% of MS 222, and dissected. On the whole, 66 experimentally infected and 96 control fish were killed. The experiments were conducted with permission of the Lab-Animal Care and Use Committee of the University of Jyväskylä (licence number 30/30.5.2005).

## Results

Upon release into experimental tanks fish frequently visited all three compartments. After 30-40 min this activity decreased and preference for the dark compartment was observed. This was accompanied by aggressive interactions, which occurred almost exclusively over the dark substrate. By the end of the first hour, distribution of the fish stabilized. Occurrence of fish in the dark compartment at 1 hour after release did not differ from that at 3 hours (Two-way ANOVA, *P *= 0.577 for control fish, *P *= 0.215 for infected fish). Thus, the 1 and 3 hours data were pooled in the experiment on substrate choice.

### Aggression Rate

**Figure 1:**
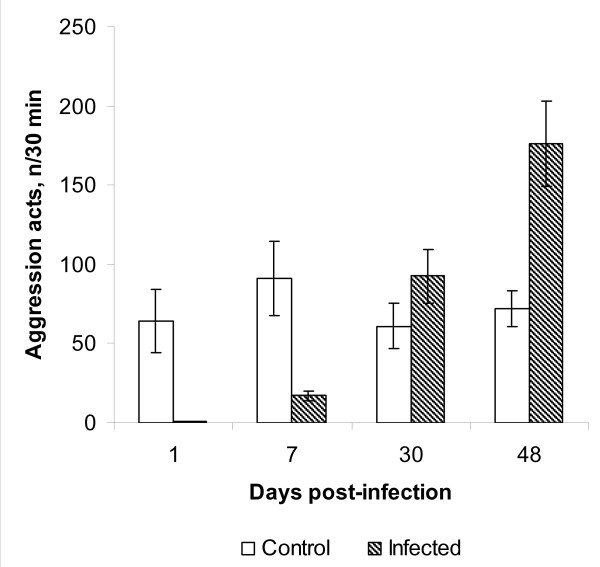
**Aggression rate (mean ± SE number of aggression acts 30 min^-1^) of juvenile *Oncorhynchus mykiss *in the course of development of *Diplostomum spathaceum *metacercariae**. N = 12.

Aggression rate changed with the age of infection (Two-way ANOVA: *F *= 28. 957, *df *= 3, *P *< 0.001; Fig. 1) and differed between infected and control fish (Two-way ANOVA: *F *= 15.553, *df *= 1, *P *< 0.001; Fig. 1). There was a significant interaction between infection status and time in the aggression rate of fish (Two-way ANOVA: *F *= 26.911, *df *= 3, *P *< 0.001). Post hoc tests revealed that, in control rainbow trout, the aggression level did not change with time from 1 to 48 days (*P *> 0.997 in all cases, Fig. 1). In contrast, aggression rate of experimentally infected rainbow trout increased during the course of the experiment. Post hoc tests indicated that in experimentally infected fish the aggressiveness was significantly lower on day 1 than on the other days (*P *< 0.001 in all cases, Fig. 1). In addition, the number of aggressive acts increased among the infected fish significantly from day 7 to days 30 and 48 PI (*P *< 0.001 in both cases), while the increase from day 30 to day 48 was not significant (*P *= 0.087). Post hoc comparisons between experimentally infected and control fish indicated a significantly lower aggression rate in infected fish on days 1 and 7 PI (*P *< 0.001 and *P *= 0.003, respectively). There were no differences on day 30 (*P *= 0.075), but infected fish became significantly more aggressive than control fish on day 48 PI (*P *= 0.027, Fig. 1).

### Substrate Choice

**Figure 2:**
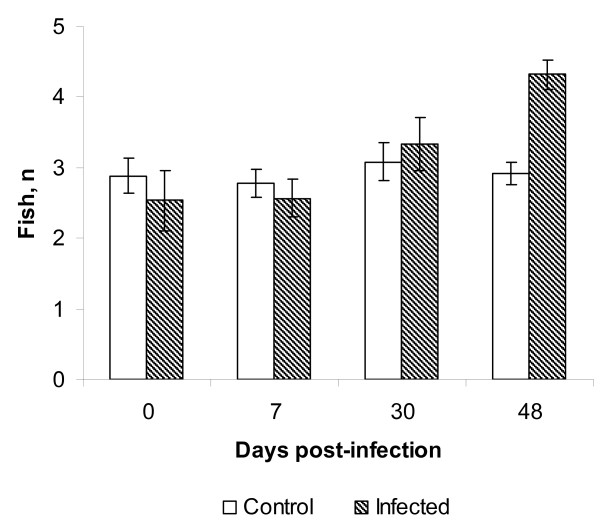
**Preference for the dark substrate estimated as the mean ± SE number of juvenile *Oncorhynchus mykiss *in the compartment with the dark bottom**. N = 12.

More fish occurred in the compartment with the dark bottom (mean ± SE: 2.9 ± 0.1 for control, 3.2 ± 0.2 for experimentally infected fish) than in the compartment with the white bottom (mean ± SE: 1.1 ± 0.1 for control, 0.7 ± 0.1 for experimentally infected fish). There was no difference between infected and control fish when the data were pooled over the whole experimental period (Two-way ANOVA: *F *= 2.112, *df *= 1, *P *= 0.150). Preference for the dark compartment increased in the course of the infection (Two-way ANOVA: *F *= 5.793, *df *= 3, *P *= 0.001). There was a significant interaction between infection status and time in the experiments on substrate choice of the fish (Two -way ANOVA: *F *= 4.556, *df *= 3, *P *= 0.005). Post hoc tests indicated an increase in preference for the compartment with a dark bottom in experimentally infected fish during the development of infection, so that while days 1 and 7 PI did not differ from each other (*P *= 0.930) they both differed from day 30 (*P *= 0.034 and *P *= 0.043, respectively) and day 48 PI (*P *< 0.001 in both cases, Fig. 2). There was also a significant difference between days 30 and 48 PI (*P *= 0.010; Fig. 2). At the same time, post hoc tests suggested that the preference for the dark substrate in control fish remained at the same level throughout the experiment (*P *> 0.423 in all cases, Fig. 2). Post-hoc comparisons between experimentally infected and control rainbow trout showed no significant differences between the groups (*P *> 0.350 in all cases) before day 48 when the experimentally infected fish demonstrated a higher preference for the dark substrate than the control fish (*P *< 0.001, Fig. 2).

### Dyadic Contests

Out of sixteen trials, the infected fish lost twelve contests while the controls lost only two contests and two ended draw (Table 2). Thus the experimentally infected rainbow trout lost significantly more contests than the control fish (Chi-square test: *X*^2^_1 _= 7.143, *P *= 0.008). The mean length of winners did not differ from the length of losers (Mann-Whitney *U *test: *U *= 90, N_1 _= N_2 _= 14, *P *= 0.713). Of 14 resolved contests, larger fish won in 8 trials, smaller in 5 trials and in one trial fish were of equal length (Chi-square test: *X*^2^_1 _= 0.643, *P *= 0.423).

**Table 2:** Mean (± SE) length, mean (± SE) intensity of infection and results of dyadic contests between experimentally infected and control rainbow trout

Fish infection state	Fork length, mm	Intensity of infection	Winners	Losers	Draw
Experimentally infected	119.2 ± 1.1	87.9 ± 5.8	2	12	2

Control	120.3 ± 1.2	5.7 ± 0.6	12	2	2

In dyadic contests between control fish with low parasite load (mean ± SE: 5.7 ± 0.6 parasites fish^-1^), the dominants were significantly larger than subordinates (Mann-Whitney *U *test: *U *= 14.5, N_1 _= N_2 _= 12, *P *< 0.001). No difference in infection rate between losers and winners was observed (Mann-Whitney *U *test: *U *= 46.5, N_1 _= N_2 _= 12, *P *= 0.141).

## Discussion

Parasite-induced alterations in the phenotype and behaviour of intermediate hosts that would increase vulnerability to predation by definitive hosts benefit trophically transmitted parasites (parasite manipulation hypothesis) [1,38,2,24]. Cryptic behaviour together with territoriality, on the other hand, reduces vulnerability to predation, e.g., [39,6,22], thus decreasing parasite transmission. The present results are consistent with the idea that *Diplostomum spathaceum *induces such behavioural changes in the fish hosts that increase the probability of predation and thereby increase transmission to the definitive hosts. First, the experimentally infected rainbow trout decreased their aggressiveness (which should decrease risk of predation) immediately after infection when parasites were not yet infective. However, the aggression rate of the experimentally infected fish increased during the course of infection so that when the parasite larvae were fully developed to infect the definitive hosts, the aggressiveness of infected fish exceeded that of controls. Second, quite surprisingly, in spite of their increased aggressiveness, the experimentally infected fish lost contests against the control fish at the time when the parasites became fully infective. Thus, the results indicate that the parasitized fish are aggressive but loose fights for a territory, thereby paying the cost of aggressiveness without the benefit of acquiring a territory. Both of these behaviours should increase transmission of the parasites as expected by the parasite manipulation hypothesis. Alternatively, the behavioural changes may be side-effects of infection [2-4], as the newly infected fish could reduce their activity because of the stress caused by parasite penetration. Later, the irritation caused by the grown metacercariae and products of their metabolism could induce increased aggressiveness.

Definitive hosts of *D. spathaceum *are fish-eating birds. The current behavioural changes of host fish associated with development of the parasite do not necessarily increase predation risk by birds only, but could lead to increased predation also by non-host predators, especially by piscivorous fish. However, Seppälä et al. [40] did not find any difference in non-host predation of infected and control fish. Moreover, a recent modelling study indicates that non-host predation should not constrain the evolution of parasite manipulation. When the "normal" predation risk is not very high, adaptations that lead to a general increase of predation should be favoured by selection even though they would expose the hosts to increased predation risk also by non-suitable hosts [41]. Indeed, together with impaired crypsis, lowered escape behaviour, reduced shoaling behaviour, higher catchability [24,22,25] and surface-seeking behaviour [23] of *D. spathaceum*-infected fish, the present behavioural changes indicate an ability of *D. spathaceum *to manipulate the phenotype of its intermediate hosts in several ways to increase parasite transmission to the definitive hosts.

Changes in aggressive behaviour, substrate choice and contestability observed in this study depended on the level of infection. Enhanced aggression rate and impaired contestability developed only in heavily infected fish. In fish with a low level of *D. spathaceum *infection (control fish), outcome of contests did not depend on the intensity of infection, but only on the size of the combatants. Even small differences in fish size allowed a larger fish to out-compete a smaller opponent as previously shown on other salmonid fishes [15,34]. The level of experimental infection used in our study was similar to that in other host-manipulation experiments [21,25] and was well within the limits of that in naturally infected fish [36,37]. In nature, *D. spathaceum *cercariae are distributed unevenly [42] and only a fraction of fish population would be heavily parasitized. Competition for a suitable territory would include different combinations of contests: symmetrical, when both combatants are either with low or high infection, and asymmetrical when the combatants possess different parasite loads [43]. The transmission of the parasites to the definitive hosts is expected to increase in symmetrical contests when the opponents are heavily infected and demonstrate high aggression that makes fish less vigilant and more conspicuous to visual predators [10,28]. Efficient transmission could also be expected in asymmetrical contests where heavily infected and highly aggressive combatants lose fights for a safe territory. In symmetrical contests between fish with low infection, the conflict is supposed to be resolved rapidly if the opponents are different in size [15] or prior residency [29]. In this case, both opponents would be less exposed to predators because the winner gets safe territory and both do not fight for too long.

Natural habitats of juvenile salmonids are heterogeneous shallow-water areas which vary in substrate colouration and availability of shelters [39,6]. The sites where fish are cryptic and safe are contestable [6,34]. In experiments on cryptic behaviour of *Oncorhynchus mykiss *both infected with *D. spathaceum *and control fish preferred dark background to white [22]. We also recorded both infected and control fish more often over dark than over light background, and infected fish demonstrated growing preference for a dark substrate. In the experiments of [22], a reduction of the preference for the dark-substrate in infected fish (8 months PI) was most probably related to cataract formation, which is time-dependent [21]. Our experiments were terminated before the intensive cataract formation. Moreover, we did not test individual fish as did Seppälä et al. [22], but groups of 5 fish among which aggressive interactions modified the preference for dark substrate. Thus, by the time when metacercariae were infective to the definitive hosts, experimentally infected as well as control fish showed a pronounced preference for dark substrate and aggressiveness that provoked fighting for individual territories.

What are the mechanisms behind the increased aggressiveness but reduced contest ability of infected fish? Aggressive individuals are usually winners in contests for a territory [15,5,6]. Variability in aggression rate is affected by a number of factors including parasite load. In the study of trematode infection and behaviour of upland bullies *Gobiomorphus breviceps*, no influence of trematode *Telogaster opisthorchis *metacercariae on aggression and dominance was found [12]. Variations in parasite-mediated changes in aggression of hosts could result from different localization of parasites within the host body and different effects on hormonal regulations of fish behaviour [44]. Trematode metacercariae often impair sensory function and the control of swimming behaviour that causes reductions in swimming speed, detection and manoeuvre ability [23,45-47]. Fast growing metacercariae of *D. spathaceum *concentrate in the eye-lenses, thus directly influencing vision, the main sensory modality in fish behaviour. The effect is especially strong after cataract formation [21]. In contests among experimentally infected fish, highly aggressive individuals with well-developed metacercariae (30 to 48 days PI) often missed their opponents and sometimes bumped into the walls of the tank. This suggests that infected fish are less precise and even being more aggressive can hardly win territorial contests with uninfected conspecifics. Size and weight of combatants were very similar and did not affect the outcome of the dyadic contests in our study. Another possible explanation for losing combats is that fish can assess fighting ability or "resource holding potential" [43] of the opponent and adjust aggression levels accordingly. Salamanders with high load of parasites were more aggressive in contests against similarly infected opponents, than in asymmetric contests against opponents with low parasite load [48].

Changes in aggression rate and contestability could be regarded either as host manipulation or by-products of infection, or a combination of both. Vision impaired by growing parasites makes fish less capable combatants against opponents with intact vision. When vision of both opponents is impaired, the contest cannot be resolved easily and aggression escalates. Symmetrical contests usually last longer than asymmetrical ones [43]. Thus, impaired vision could cause both reduced contestability and increased aggression. However, we observed suppressed aggression of infected fish at the beginning of infection. At this time vision was not impaired [21], (and present results) that suggests that parasite-induced hormonal effects may be involved in the control of aggression. In this case, aggression could be considered either a manipulated trait, or a side-effect of pathology caused by penetration of parasites. As to the reduced contestability, it could be just a byproduct of impaired vision.

## Conclusions

Our results agree with the idea that *Diplostomum spathaceum *modify fish behaviour in a way that increases the probability of predation and transmission to the definitive hosts. The aggression rate of the experimentally infected fish increased during the course of infection so that when the parasite larvae were infective, the aggressiveness of infected fish exceeded that of controls. In spite of their increased aggressiveness, the infected fish lost contests against the control fish at the time when the parasites became fully infective. Thus, the parasitized fish are aggressive but lose fights for a territory, thereby paying the cost of aggressiveness without the benefit of acquiring a territory. The parasite-associated changes in social interactions could increase the net virulence of the parasite beyond that observed in traditional laboratory tests where the impacts of parasites are studied without taking into account the social interactions between host individuals.

## Competing interests

The authors declare that they have no competing interests.

## Authors' contributions

VM and AP conceived the study, performed the experiments, and wrote the manuscript. JT advised on the statistical analysis and clarified the manuscript. TV conceived and supervised the study. All authors read and approved the final manuscript.
